# Targeting tumor cell-derived CCL2 as a strategy to overcome Bevacizumab resistance in ETV5^+^ colorectal cancer

**DOI:** 10.1038/s41419-020-03111-7

**Published:** 2020-10-24

**Authors:** Haoran Feng, Kun Liu, Xiaonan Shen, Juyong Liang, Changgang Wang, Weihua Qiu, Xi Cheng, Ren Zhao

**Affiliations:** 1 Department of General Surgery, Ruijin Hospital, School of Medicine, Shanghai Jiao Tong University, 200025 Shanghai, China; 2 Shanghai Institute of Digestive Surgery, Ruijin Hospital, School of Medicine, Shanghai Jiao Tong University, 200025 Shanghai, China; 3 Department of General Surgery, Ruijin Hospital North, School of Medicine, Shanghai Jiao Tong University, 201800 Shanghai, China; 4 Division of Gastroenterology and Hepatology, Renji Hospital, School of Medicine, Shanghai Jiao Tong University, 145 Middle Shandong Road, 200001 Shanghai, China

**Keywords:** Cancer therapeutic resistance, Chemotherapy

## Abstract

In our previous study, ETV5 mediated-angiogenesis was demonstrated to be dependent upon the PDGF-BB/PDGFR-β/Src/STAT3/VEGFA pathway in colorectal cancer (CRC). However, the ability of ETV5 to affect the efficacy of anti-angiogenic therapy in CRC requires further investigation. Gene set enrichment analysis (GSEA) and a series of experiments were performed to identify the critical candidate gene involved in Bevacizumab resistance. Furthermore, the ability of treatment targeting the candidate gene to enhance Bevacizumab sensitivity in vitro and in vivo was investigated. Our results revealed that ETV5 directly bound to the VEGFA promoter to promote translation of VEGFA. However, according to in vitro and in vivo experiments, ETV5 unexpectedly accelerated antiVEGF therapy (Bevacizumab) resistance. GSEA and additional assays confirmed that ETV5 could promote angiogenesis by inducing the secretion of another tumor angiogenesis factor (CCL2) in CRC cells to facilitate Bevacizumab resistance. Mechanistically, ETV5 upregulated CCL2 by activating STAT3 to facilitate binding with the CCL2 promoter. ETV5 induced-VEGFA translation and CCL2 secretion were mutually independent mechanisms, that induced angiogenesis by activating the PI3K/AKT and p38/MAPK signaling pathways in human umbilical vein endothelial cells (HUVECs). In CRC tissues, ETV5 protein levels were positively associated with CD31, CCL2, and VEGFA protein expression. CRC patients possessing high expression of ETV5/VEGFA or ETV5/CCL2 exhibited a poorer prognosis compared to that of other patients. Combined antiCCL2 and antiVEGFA (Bevacizumab) treatment could inhibit tumor angiogenesis and growth more effectively than single treatments in CRCs with high expression of ETV5 (ETV5^+^ CRCs). In conclusion, our results not only revealed ETV5 as a novel biomarker for anti-angiogenic therapy, but also indicated a potential combined therapy strategy that involved in targeting of both CCL2 and VEGFA in ETV5^+^ CRC.

## Introduction

Colorectal cancer (CRC) is one of the most common cancers worldwide, and its morbidity and mortality rank third among all cancers^1^. Despite advancements in the diagnosis and treatment of CRC over the past few decades, the prognosis for this disease remains poor^2^. Angiogenesis is a hallmark process in the oncogenesis of CRC^3–5^, and vascular endothelial growth factor A (VEGFA) and its receptors (VEGFR-1/VEGFR-2) play dominant roles in the regulation of this complex process. Attenuation of VEGF-VEGFR signaling can disrupt vascularization, and this has been considered as a promising therapeutic strategy for CRC^6^.

Bevacizumab, a clinically used anti-angiogenic drug, can specifically target VEGFA to inhibit VEGF–VEGFR signaling^6^. A combination of Bevacizumab and chemotherapy is the first-line treatment for metastatic CRC^6–8^. However, some CRC patients are resistant to Bevacizumab, and the overall response rate is limited^9^. Therefore, exploration of mechanisms of resistance to anti-angiogenic treatments will be beneficial for identification of potential targets, that can be exploited to overcome Bevacizumab resistance in CRC.

The ETS transcription factor family contains 28 factors and can be divided into 12 subfamilies^10^. E26 transformation-specific variant 5 (ETV5), a member of the ETS family, has been reported to be involved in the progression of hematologic cancer, endometrial cancer^11,12^, ovarian cancer^13,14^, prostate cancer, and thyroid cancer^15,16^. Previous studies have also revealed that members of the ETS family can trigger angiogenesis in multiple tumors^13,17,18^. Similarly, our previous study demonstrated that ETV5 facilitated CRC angiogenesis via the PDGF-BB/Src/STAT3/VEGFA signaling pathway^19^. Additionally, chemokine signaling events such as CCL2/CCR2 signaling and CXCL11/CXCR7 signaling have been reported to play critical roles in tumor angiogenesis^20,21^. Paracrine chemokine signaling also represents a critical drug resistance mechanism in cancers^22^. ETV5 has been reported to regulate the expression of chemokines such as Ccl7, Ccl9, and Ccl12 in Sertoli cells in mice. However, the ability of ETV5 to regulate antiVEGF therapy sensitivity and the underlying mechanisms require further clarification.

In the present study, we found that ETV5 could promote Bevacizumab resistance via the secretion of CCL2 and CCL2, could also induce angiogenesis by activating the PI3K/AKT and p38/MAPK signaling pathways in human umbilical vein endothelial cells (HUVECs). A combination of Bevacizumab and antiCCL2 treatment exerted a synergistically inhibitory effect on tumor growth and angiogenesis. Our results indicated that targeting both CCL2 and VEGFA might provide a promising and effective therapeutic approach for the treatment of ETV5^+^ CRC.

## Materials and methods

### Patient specimens

The tumor tissues and adjacent normal tissues used in this study were collected from 75 CRC patients who underwent surgery at Ruijin Hospital from 2010 to 2011. The information regarding these 75 patients has been described previously^23^. Informed consent was obtained from all patients.

### Cell lines and therapeutic antibodies

The human CRC cell lines RKO and HT29 were purchased from American Type Culture Collection (ATCC). HUVECs were purchased from Shanghai Institutes for Biological Sciences, Chinese Academy of Sciences. All cells were cultured in RPMI 1640 supplemented with 10% fetal bovine serum (FBS) and antibiotics at 37 °C with 5% CO_2_. Bevacizumab (a humanized monoclonal antibody that specifically binds to all VEGFA isoforms with high affinity) was purchased from MedChemExpress (Cat. No. HY-P9906), and it was added to the indicated cells at concentrations of 250 μg/mL. Tumor-bearing nude mice were treated with Bevacizumab at a dose of 2 mg/kg every three days. Anti-CCL2 (clone 2H5) antibody, an IgG monoclonal antibody that neutralizes the bioactivity of human natural or recombinant CCL2, was purchased from BioLegend (Cat. No. 505913), and it was added to the indicated cells at a concentration of 10 ng/mL. Tumor-bearing nude mice were treated with antiCCL2 at a dose of 2 mg/kg every 3 days. Recombinant human VEGF 165 protein was purchased from Abcam (ab83572) systems. Recombinant human CCL2 protein was purchased from Invitrogen (RP-75662). A STAT3 inhibitor (C188-9) was purchased from Selleck (Cat. No. S8605), and it was added to the indicated cells at a concentration of 5 μΜ. A STAT3 activator (Colivelin) was purchased from Santa Cruz Biotechnology (Sc-361153), and it was added to the indicated cells at a concentration of 0.5 μΜ.

### RNA extraction and quantitative RT-PCR

Total RNA was extracted from HT29/Control and HT29/shETV5 cells using TRIzol (Invitrogen, USA) according to the manufacturer’s instructions. The cDNA was synthesized using a reverse transcription kit (Invitrogen, CA). Quantitative PCR was performed using TaqMan® Gene Expression Assays (Thermo Fisher Scientific). The primers used were: CXCL11: (forward) 5′-GACGCTGTCTTTGCATAGGC-3′, (reverse) 5′-GGATTTAGGCATCGTTGTCCTTT-3′; CXCL5, (forward) 5′-GAGAGCTGCGTTGCGTTTGTTTAC-3′, (reverse) 5′-CCGTTCTTCAGGGAGGCTACCACT-3′; CCL2, (forward) 5′-CAGCCAGATGCAATCAATGCC-3′, (reverse) 5′-TGGAATCCTGAACCCACTTCT-3′; CCL13, (forward) 5′-TGCTGACCCAAAGGAGAAG-3′, (reverse) 5′-GCCAGAGGAGAATGGAAAAG-3′.

### Western blot analysis

Cells were harvested and lysed in the RIPA buffer in the presence of a Protease Inhibitor Cocktail (Pierce, USA) and a Protein Phosphatase Inhibitor Cocktail (New Cell & Molecular Biotech, China). One hundred micrograms of protein were separated by 10% SDS-PAGE gel and transferred onto PVDF membranes (Tanon, China). The membranes were blocked with 5% bovine serum albumin (BSA) for 2 h and then incubated overnight with primary antibodies at 4 °C. The blots were probed with anti-STAT3 (9139S, CST, 1:1000, Boston, USA), anti-p-STAT3 (9145S, CST, 1:1000, Boston, USA), anti-p-P38 (4511S, CST, 1:1000, Boston, USA), anti-P38 (9212S, CST, 1:1000, Boston, USA), anti-VEGFR2 (9698S, CST, 1:1000, Boston, USA), anti-p-VEGFR2 (2478S, CST, 1:1000, Boston, USA), anti-AKT (4691S, 1:1000, CST, Boston, USA), anti-p-AKT (4060S, CST, 1:1000, Boston, USA), anti-CCL2 (2027S, 1:1000, CST, Boston, USA), and anti-GAPDH (ab8245, Abcam, 1:10,000, Cambridge, UK). GAPDH was used as the internal control. Goat antimouse or goat antirabbit horseradish-peroxidase-conjugated IgG was used as the secondary antibody (Santa Cruz Biotechnology). The membranes were incubated with secondary antibody for 2 h at room temperature, and bands were visualized using an enhanced chemiluminescence detection system (Amersham Bioscience, Piscataway, NJ, USA) according to the manufacturer’s instructions.

### Generation of cells with gene overexpression and knock-down

Lentiviruses for ETV5 overexpression and knock-down were purchased from Shanghai Bioegene Co., Ltd. (Shanghai, China). Lentiviral particles were transduced into CRC cells according to the manufacturer’s instructions, and this was followed by stable selection. Lentivirus transfection was performed as described previously^19^. The antibiotic puromycin (2 μg/mL) was used to select stably transfected cells. The effects of overexpression and knock-down were evaluated by western blotting.

### Cell viability assays

Approximately 3000 HUVECs were plated into 96-well plates and cultured in a 37 °C/5% CO_2_ incubator as per methods described previously^19^. Then, the supernatants from RKO and HT29 cells were added. Using a CCK-8 assay (Dojindo Molecular Technologies Inc.), cell viability was assessed at the 1st, 2nd, 3rd, 4th, and 5th day.

### Endothelial tube formation analysis

HUVECs were treated with supernatants from RKO and HT29 cells in 96-well plates at a density of 1 × 10^4^ cells per well at 37 °C. Each plate was pre-coated with 50 μL of Matrigel (BD Bioscience) at 37 °C for 30 min. After 6 h of incubation, tubules were observed by microscopy and analyzed using the Image-Pro Plus software.

### Chick embryo chorioallantoic membrane (CAM) assay

The CAM assay was performed as per previously described methods^3^. Briefly, filter paper was placed on eggs with a round window cut in the egg shell in advance, and then, 30 μL of the cell culture supernatant from RKO and HT29 cells was added dropwise onto the filter paper tray. The egg was then sealed with a transparent tape for 3 days. On the 10th day, the eggs were imaged using a MacroPATH dissecting microscope (Milestone, Italy), and the number of blood vessels surrounding the filter paper tray was counted.

### In vivo xenograft tumor model

A total of 35 male nude mice (4 weeks old; Institute of Zoology, Chinese Academy of Sciences) were used for this study. CRC cells (1 × 10^6^ cells) were subcutaneously injected into the mice, and tumor nodules were measured every 5 days using a vernier caliper. The tumor volume was calculated using the following formula: *V* = *π*/6 × (*W*^2^ × *L*). Therapeutic antibodies were dissolved in NS (normal saline solution), and they were administered via tail vein injection every three days (Bevacizumab: 2 mg/kg, anti-CCL2 antibody: 2 mg/kg). The mice were euthanized 30 days after the first injection. The tumors were weighed and fixed in formalin. All steps were performed according to the Guide for the Care and Use Laboratory Animals of Ruijin Hospital, Shanghai Jiao Tong University School of Medicine.

### Chromatin immunoprecipitation (ChIP)

ChIP assays were performed in RKO cells following the chromatin immunoprecipitation kit (Millipore) protocol as reported previously^19^. Anti-ETV5 antibodies (Santa Cruz Biotechnology, SC-22807, Dallas, USA) or anti-STAT3 antibodies (9139S, CST, 1:100, Boston, USA) were used for immunoprecipitation. After purification of the precipitated DNA, the human VEGFA promoter was amplified by qRT-PCR. The primers used to amplify the VEGFA and CCL2 promoters were: VEGFA: forward, 5′-TAGTGCTGGCGGGTAGGTTT-3′; reverse, 5′-CCAAGTTTGTGGAGCTGAGAA-3′; CCL2: 5′-TTTGGTCTCAGCAGTGAATGG-3′; reverse, 5′-AGTCAAGCAGGAGGAGGGAT-3′.

### ELISA

ELISA experiments were performed according to methods described in previous studies^24,25^. Briefly, CRC cell lines were seeded into six-well plates and incubated for 3 days. VEGFA and CCL2 expression levels in the supernatants were detected using the VEGFA Human Biotrak ELISA system (Amersham Biosciences Corp., Piscataway, NJ) and a Human MCP1 (CCL2) ELISA kit (ab179886, Abcam, Cambridge, UK), respectively.

### Luciferase reporter assay

VEGFA promoter fragments were amplified from human genomic DNA and cloned into the pGL3-Basic vector. After transfection for 72 h, Luciferase activity in RKO cells was examined using the Dual-Luciferase Assay (Promega) following the manufacturer’s instructions.

### Immunohistochemistry assay

Immunohistochemistry analysis of xenograft tumors in nude mice and CRC specimens were conducted as previously described^3,19^. The sections were incubated with antibodies against ETV5 (ab102010, Abcam, Cambridge, UK), VEGFA (ab46154, Abcam, Cambridge, UK), CD31 (3528, CST, 1:100, Boston, USA), Ki67 (1:200, Santa Cruz, Dallas, USA), and CCL2 (ab9669, Abcam, Cambridge, UK).

### Gene set enrichment analysis (GSEA) analysis

The transcription data of the raw count of 635 CRCs were downloaded from The Cancer Genome Atlas (TCGA; https://portal.gdc.cancer.gov/). The transcripts per million (TPM) for each gene were then calculated and normalized by Log2(TPM + 1). According to the median of the ETV5 gene expression values, we divided all CRCs into a High group (*n* = 212), moderate group (*n* = 211), and low (*n* = 212) group. Using GSEA software (https://www.gsea-msigdb.org/gsea/index.jsp), the enriched KEGG pathways were analyzed between the high and low groups.

### Statistical analysis

The genes that were significantly enriched in the Chemokine Signaling Pathway were displayed according to a heatmap using “pheatmap” package in R software. The gene expression values for CXCL11, CXCL5, CCL2, and CCL13 were extracted from our previous RNA-Seq data from HT29/Vector and HT29/shETV5 cells that were previously deposited into the GEO database (GSE112628). The data are expressed as the means ± SD. Analysis of variance (ANOVA) and Student’s *t*-test analyses were used for comparisons among groups. The Mann–Whitney *U*-test was used to facilitate tumor volume comparison. Categorical data were evaluated using the chi-square test or the Fisher’s exact test. ROC curves were plotted to determine the cutoff values for ETV5, VEGFA, and CCL2 expression. A log-rank test was performed to compare the survival curves of two or more groups. *P*-values of less than 0.05 were considered significant. Statistical analyses were processed using GraphPad Prism 6.0 (Inc., La Jolla, CA, United States).

## Results

### ETV5 induces Bevacizumab resistance in CRC

Our previous study revealed that ETV5 promoted CRC angiogenesis in vitro and in vivo^19^; however, the underlying mechanisms require further clarification. Here, we analyzed the promoter sequence of the VEGFA gene, and we detected a potential ETV5 binding site using JASPAR website (http://jaspar.genereg.net/) (Supplementary Fig. 1a). ChIP assays for anti-ETV5 were performed in RKO cells, and this was followed by qRT-PCR analysis of the VEGFA promoter and the upstream regions. Our results revealed that the ETV5 protein could bind to a site within the VEGFA promoter (Supplementary Fig. 1b). Moreover, the results from a luciferase reporter assay revealed higher luciferase expression in the VEGFA-promoter-ETV5 than that observed for the VEGFA-promoter-Vector (Supplementary Fig. 1c), and our findings also indicated that ETV5 could activate the wild type VEGFA promoter but not the mutant promoter (Supplementary Fig. 1d).

**Fig. 1:**
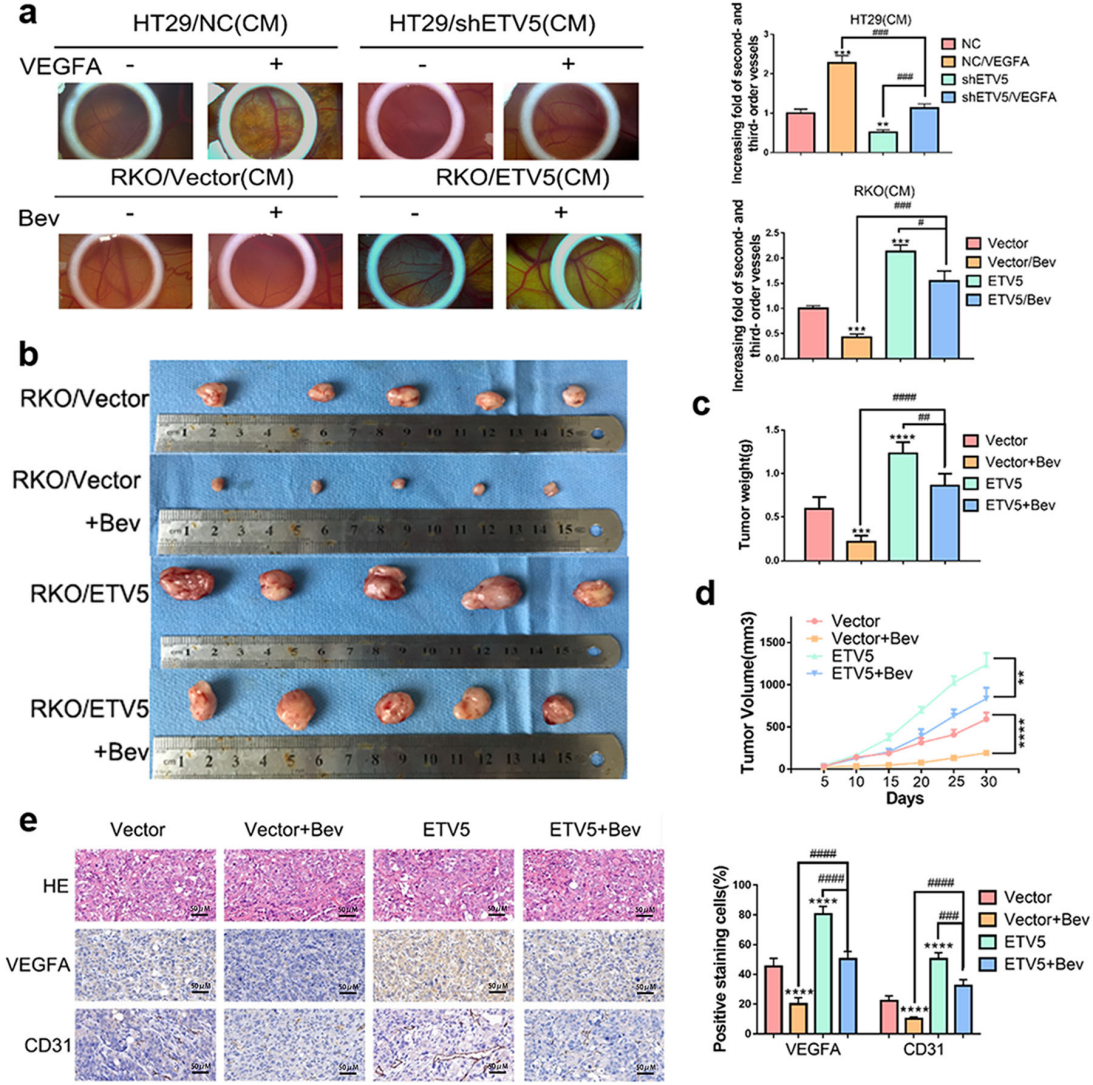
ETV5 induces Bevacizumab resistance in CRC in vivo. **a** The CAM assay was used to examine effect of Bevacizumab and VEGFA on blood vessel formation after stimulation with the supernatants from the indicated cells. Data are presented as mean ± SD of three independent experiments. **b** Tumor volumes were calculated to measure tumorigenesis ability. **c** Images of xenografted tumors after subcutaneous injection of mice with the indicated cells (*n* = 5). **d** The average tumor weight for each group was calculated. **e** Representative images of HE staining and IHC staining for CD31 and VEGFA in subcutaneous tumor tissues of the RKO/Vector, RKO/Vector+Bev(2 mg/kg), RKO/ETV5, and RKO/ETV5+Bev(2 mg/kg) groups. “*” represents in comparison with the control. Bev Bevacizumab. **p < 0.01, ***p < 0.001, ****p < 0.0001. ^##^*p* < 0.01, ^###^*p* < 0.001, ^####^*p* < 0.0001.

Our previous study found that PDGF-BB could activate VEGFA expression via the PDGFR-β/Src/STAT3 pathway in CRC^19^, and our present results indicated that ETV5 could upregulate VEGFA via transcriptional activation of VEGFA in CRC. Angiogenesis that was attenuated by ETV5 knock-down could be reversed by treatment with recombinant human VEGFA. In cancers, high expression of VEGFA was particularly related to high sensitivity to VEGFA inhibition treatment, and these cancers included liver cancer, sarcoma, and breast cancer^26–28^. However, according to the results of a CAM assay, upregulation of ETV5 actually induced Bevacizumab resistance in RKO cells (Fig. 1a). After receiving Bevacizumab treatment, RKO/ETV5 tumors grew faster than RKO/Vector tumors in vivo (Fig. 1b). The sizes of tumors in the RKO/ETV5+Bev group were larger than those of tumors in the RKO/Vector+Bev group (Fig. 1c, d). Within the tumor tissues, the RKO/ETV5+Bev group exhibited higher expression of VEGFA and CD31 than that exhibited by RKO/Vector+Bev group (Fig. 1e).

**Fig. 2:**
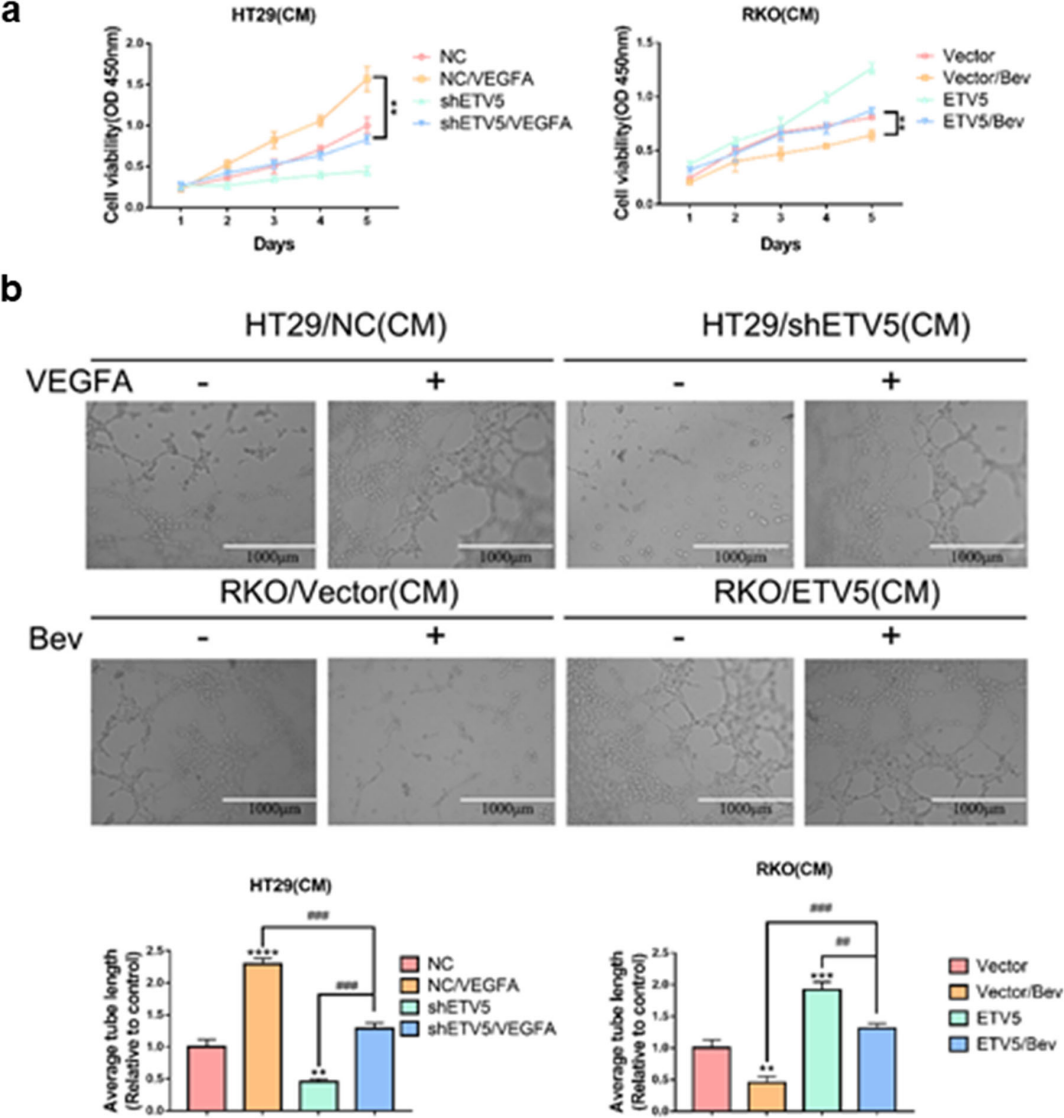
Ectopic overexpression of ETV5 promotes Bevacizumab resistance in CRC in vitro. **a** CCK-8 assay was used to analyze the effect of Bevacizumab or recombinant human VEGFA protein on proliferation of HUVECs incubated with conditioned media collected from the indicated CRC cells. **b** Representative images of the formation of HUVEC tubules incubated with supernatants collected from the indicated cells and treatment with Bevacizumab or recombinant human VEGFA protein. Bev Bevacizumab. Data are presented as mean ± SD of three independent experiments. “*” represents in comparison with the control. **p < 0.01, ***p < 0.001, ****p < 0.0001. ^##^*p* < 0.01, ^###^*p* < 0.001.

Similar results were also observed based for the CCK-8 assays and endothelial tube formation analyses. The deceased cell proliferation and tubular formation of HUVECs after ETV5 knock-down could be reversed by treatment with recombinant human VEGFA protein (Fig. 2a, b). After Bevacizumab treatment, the RKO/ETV5 group exhibited more apparent cell proliferation and tubular formation by HUVECs than that exhibited by the RKO/Vector group (Fig. 2a, b).

### CCL2 is another critical proangiogenic factor regulated by ETV5 in CRC

**Fig. 3:**
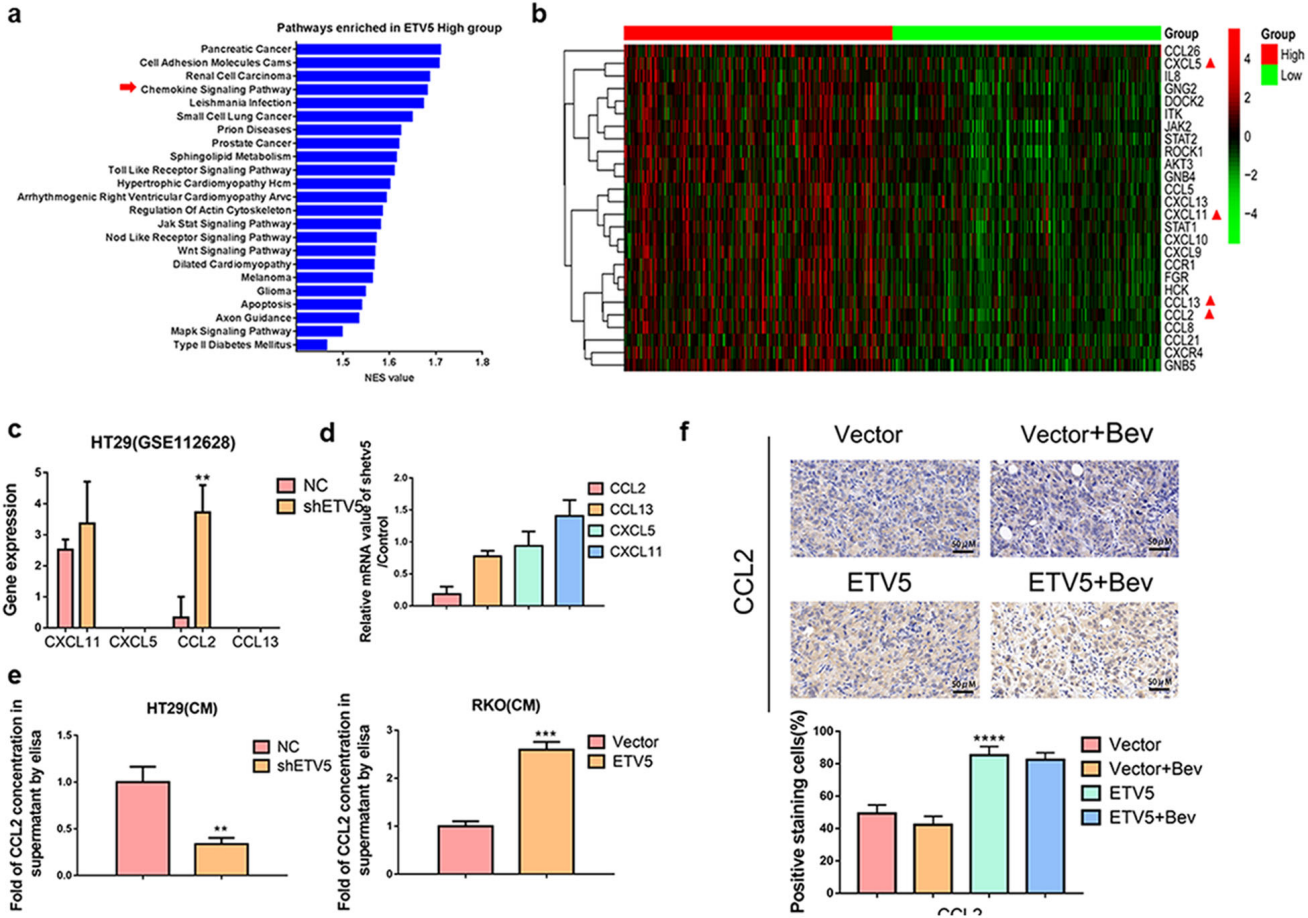
CCL2 is another pro-angiogenic factor that is regulated by ETV5 in CRC. **a** GSEA analysis revealed pathways that were positively related to ETV5 expression in CRC according to TCGA database results. **b** A heatmap was used to display the significantly enriched genes in the chemokine signaling pathway. **c** Among the significantly enriched genes, four chemokines exhibited pro-angiogenic roles (CXCL5, CXCL11, CCL2, and CCL13). The expression of these chemokines was compared between HT29/Control and HT29/shETV5 cells (GSE112628). **d** qRT-PCR was used to examine the expression of CXCL5, CXCL11, CCL2, and CCL13 in HT29/shNC and HT29/shETV5 cells. **e** CCL2 concentrations in the supernatants of HT29/Vector, HT29/shETV5, RKO/Vector, and RKO/ETV5 cells were determined by ELISA. Data are presented as mean ± SD of three independent experiments. **f** Representative images of IHC staining of CCL2 in subcutaneous tumor tissues from the RKO/Vector, RKO/Vector+Bev, RKO/ETV5, and RKO/ ETV5+Bev groups. Bev Bevacizumab. “*” represents in comparison with the control. **p < 0.01, ***p < 0.001.

The above-mentioned results indicated that ETV5 could induce Bevacizumab resistance. To further explore the potential mechanisms, we used TCGA database to perform ETV5-related GSEA analysis. According to ETV5 expression levels, we divided all CRCs into three groups that included the high group (*n* = 212), moderate group (*n* = 211), and low (*n* = 212) group. GSEA analyses were performed between the high and low groups. It should be noted that ETV5 was positively related to the activation of a chemokine signaling pathway (Fig. 3a) that was involved in angiogenesis in cancers^29,30^. In this pathway, 26 genes were significantly enriched and are further displayed according to a heatmap (Fig. 3b). Among these genes, four genes encoded proangiogenic proteins such as CXCL5, CXCL11, CCL2, and CCL13. In our previous dataset (GSE112628) that was deposited into the GEO database, only CCL2 was downregulated in HT29/shETV5 cells compared to levels in HT29/Vector cells (Fig. 3c), and this finding was further validated through the use of qRT-PCR (Fig. 3d). According to ELISA experiments, we observed higher levels of CCL2 in culture supernatants from HT29/NC cells compared to levels in supernatants from HT29/shETV5 cells, and we also observed higher levels of CCL2 in culture supernatants from RKO/ETV5 cells compared to those of supernatants from RKO/Vector cells (Fig. 3e). The expression levels of CCL2 were higher in tumor tissues in the RKO/ETV5+Bev group compared to those in the RKO/Vector+Bev group in vivo (Fig. 3f).

### ETV5 regulates CCL2 expression via STAT3 in CRC

**Fig. 4:**
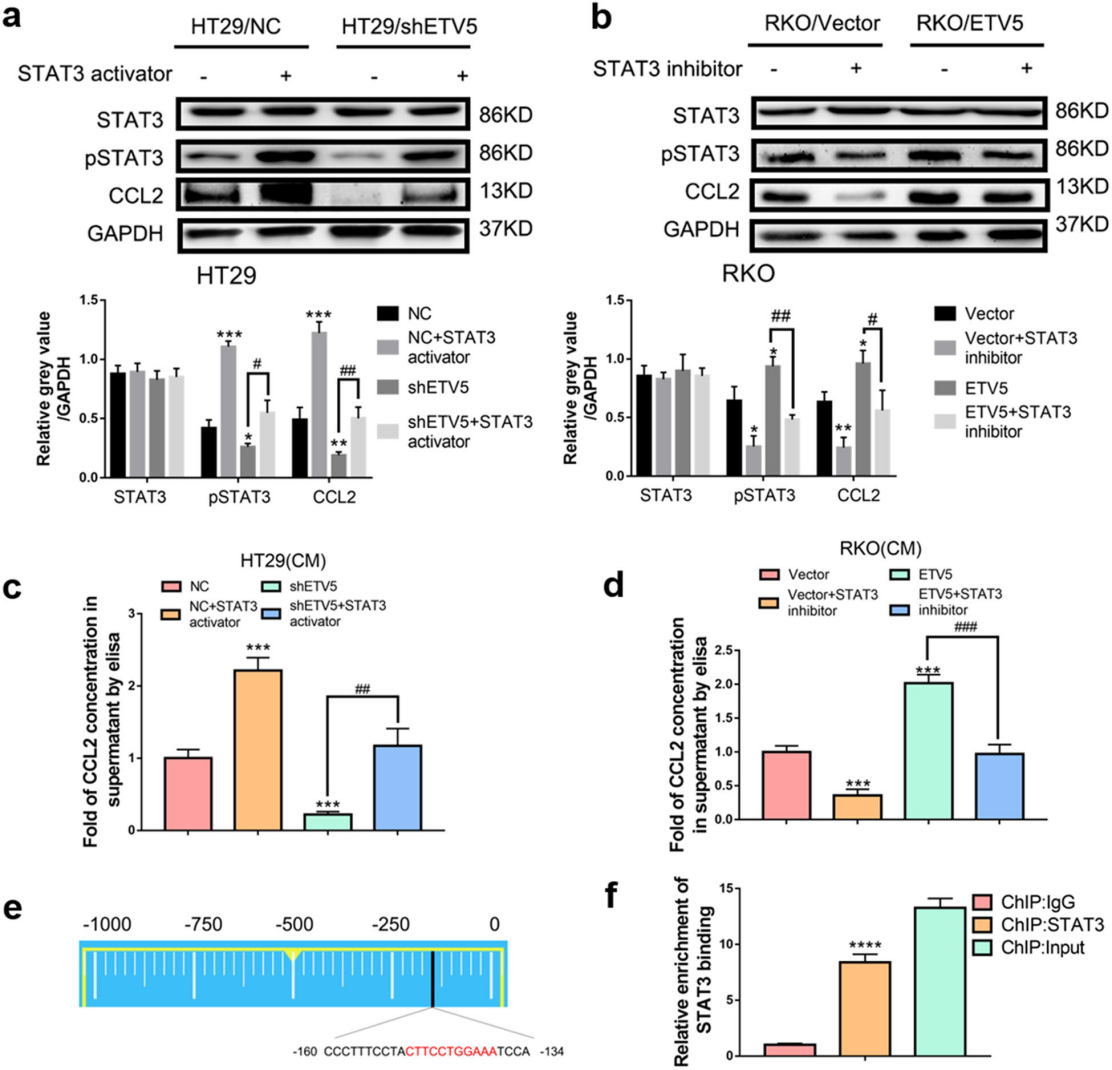
ETV5 activates STAT3-mediated transcriptional activation of CCL2 in CRC. **a**, **b** CRC cell lines were transfected with the indicated lentiviruses and treated with STAT3 inhibitor or STAT3 activator. Next, western blots were used to detect the expression of STAT3, pSTAT3, CCL2, and GAPDH. **c**, **d** ELISA analysis of CCL2 secretion after treatment with STAT3 inhibitor or STAT3 activator in ETV5 downregulated/overexpressing cells. **e** Diagram of the CCL2 promoter, where the black marker indicates the STAT3-binding sites. **f** ChIP was performed using an anti-STAT3 antibody in RKO cells to analyze STAT3 binding to the CCL2 promoter. qRT-PCR experiments were performed using primers against the indicated area in the CCL2 promoter, and the indicated region showed significant enrichment compared to that of the control. Data are presented as mean ± SD of three independent experiments. “*” represents in comparison with the control. ***p < 0.001, ****p < 0.0001, ^##^ *p* < 0.01, ^###^*p* < 0.001.

Our previous study demonstrated that ETV5 significantly promoted CRC angiogenesis through the PDGF-BB/PDGFR-β/Src/STAT3 signaling pathway^19^. Subsequent experiments were performed to explore if ETV5 regulated CCL2 expression via STAT3. First, HT29/NC and HT29/shETV5 cells were treated with a STAT3 activator, and RKO/NC and RKO/ETV5 cells were treated with a STAT3 inhibitor. Our results revealed that CCL2 expression, that was attenuated by ETV5 downregulation, was rescued by treatment with a STAT3 activator in HT29 cells (Fig. 4a, b), and ETV5-induced CCL2 secretion was also reversed by treatment with a STAT3 inhibitor in RKO cells (Fig. 4c, d). Interestingly, we found that STAT3 was likely to function as a transcription factor of CCL2 according to the JASPAR website (http://jaspar.genereg.net/) (Fig. 4e). Furthermore, ChIP assays were performed in RKO cells, and this was followed by qRT-PCR of the CCL2 promoter and the upstream regions. The results reveal that STAT3 binds to the CCL2 promoter (black marker positions; Fig. 4e, f).

### CCL2 partially contributes to ETV5-mediated angiogenesis in CRC

**Fig. 5:**
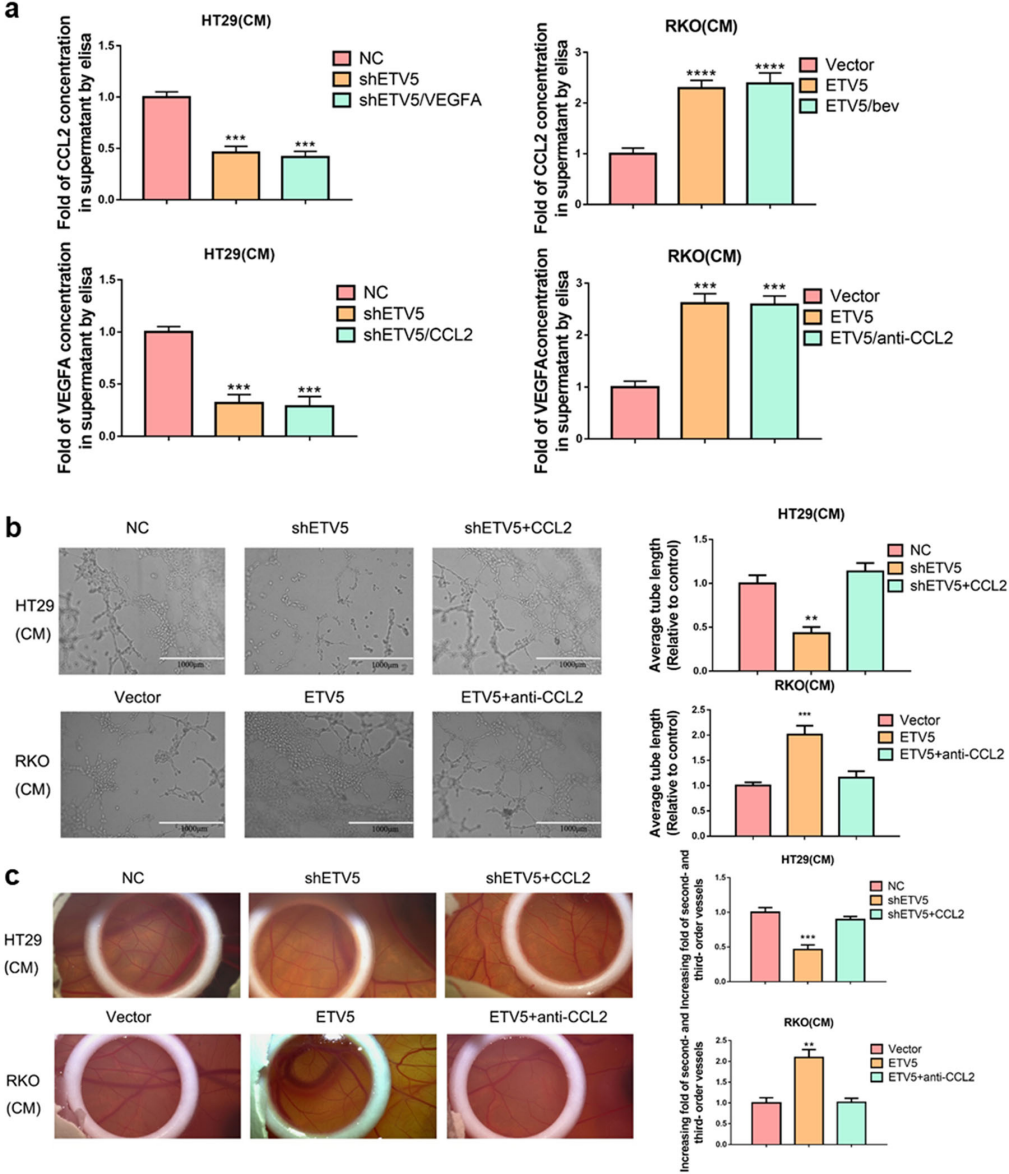
Tumor cell-derived CCL2 partially contributes to ETV5-mediated angiogenesis in CRC. **a** ELISA analysis of CCL2 and VEGFA secretion after treatment with recombinant human VEGFA, CCL2, Bev, or anti-CCL2 in ETV5 downregulated/overexpressing cells. Bev Bevacizumab. **b** Representative images of the formation of HUVEC tubules following incubation with supernatants collected from the indicated cells and treatment with recombinant human CCL2 protein or CCL2 antibody. **c** The CAM assay was used to examine the effect of recombinant human CCL2 protein or CCL2 antibody on blood vessel formation after stimulation with the supernatants from the indicated cells. Data are presented as mean ± SD of three independent experiments. “*” represents in comparison with the control. **p < 0.01, ***p < 0.001, ****p < 0.0001.

We found that the attenuated secretion of CCL2 caused by ETV5 downregulation was not rescued by administration of recombinant human VEGFA in HT29 cells, and ETV5 upregulation-induced CCL2 secretion was also not reversed by administration of Bevacizumab in RKO cells (Fig. 5a). Attenuated VEGFA secretion resulting from ETV5 downregulation was not rescued by administration of recombinant human CCL2 protein in HT29 cells, and ETV5 upregulation-induced VEGFA secretion was also not reversed by administration of anti-CCL2 in RKO cells (Fig. 5a). According to endothelial tube formation analysis and CAM assays, ETV5 upregulation-induced tubular formation in HUVECs could be reversed by treatment with an anti-CCL2 antibody, and ETV5 knock-down-attenuated tubular formation in HUVECs could be rescued by the addition of recombinant human CCL2 protein (Fig. 5b,c). These results indicated that ETV5 promoted angiogenesis by enhancing CCL2 secretion, and this process was independent of VEGFA.

### Combination treatment using antiVEGFA and antiCCL2 results in a synergistic antitumor effect in CRC

**Fig. 6:**
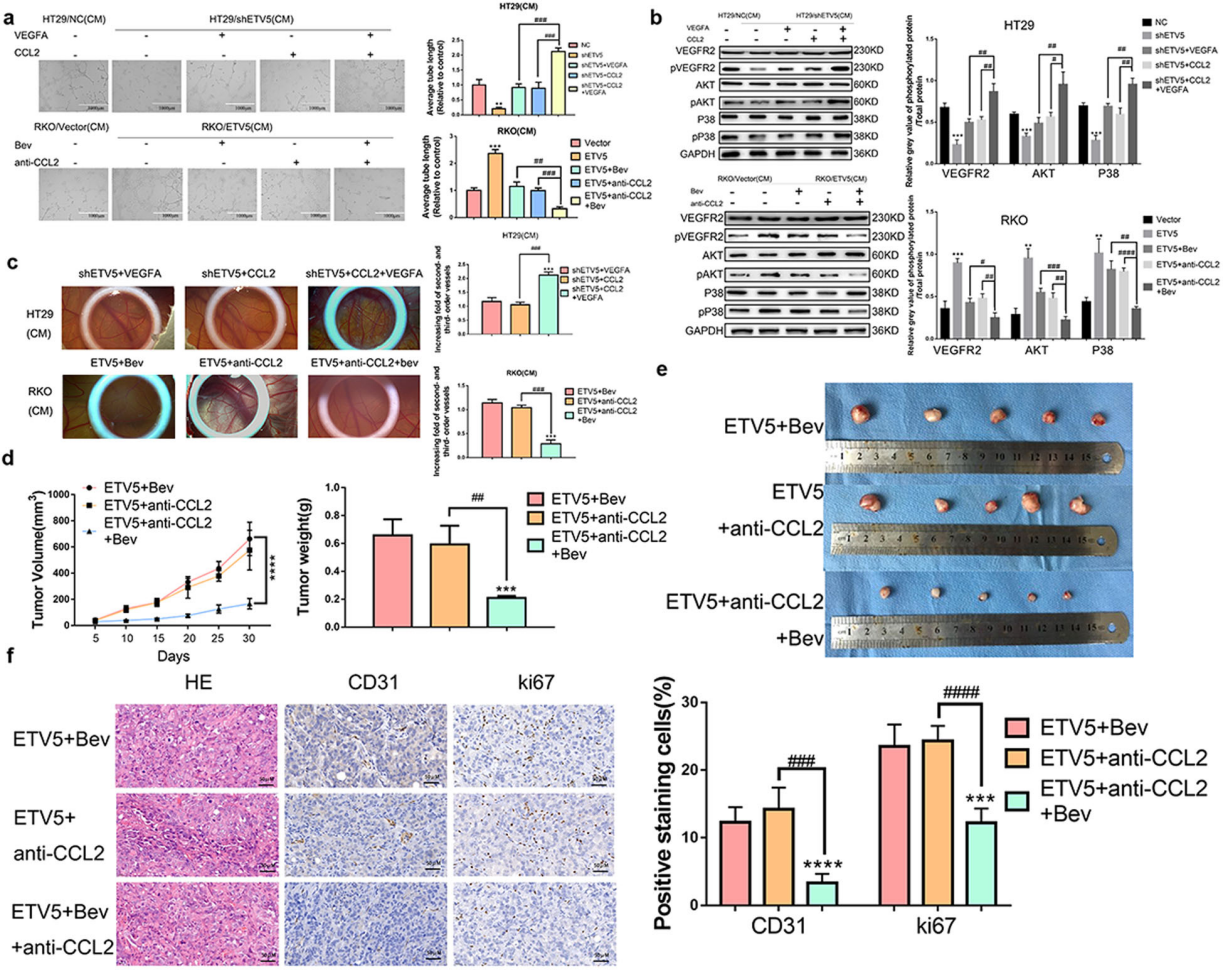
Combination treatment using antiVEGFA and antiCCL2 synergistically inhibits tumor growth and angiogenesis in CRC. **a** Representative images of the formation of HUVEC tubules following incubation with supernatants collected from the indicated cells and treatment with recombinant human VEGFA and CCL2 protein or Bev and antiCCL2. Data are presented as mean ± SD of three independent experiments. **b** HUVECs were incubated with the indicated supernatants and treated with recombinant human VEGFA and CCL2 protein or Bev and antiCCL2. Activation of the VEGFR downstream signaling pathway was determined according to western blotting. GAPDH was used as a loading control. **c** The CAM assay was used to examine effect of a combination treatment using recombinant human VEGFA and CCL2 protein or a combination of Bevacizumab and antiCCL2 on blood vessel formation after stimulation with the supernatants from the indicated cells. Data are presented as mean ± SD of three independent experiments. **d** Tumor volumes were calculated to measure tumorigenesis ability of RKO/ETV5 cells after receiving antiVEGFA or/and antiCCL2 treatment. **e** Images of xenografted tumors derived from RKO/ETV5 cells in every group. The average tumor weight for each group (*n* = 5) was calculated. **f** Representative images of HE stains and IHC staining for CD31 and ki67 in subcutaneous tumor tissue in the RKO/ETV5, RKO/ETV5+Bev, and RKO/ETV5+Bev+anti-CCL2 groups. Bev Bevacizumab. “*” represents in comparison with the control. **p < 0.01, ***p < 0.001, ****p < 0.0001. ^##^*p* < 0.01, ^###^*p* < 0.001, ^####^*p* < 0.0001.

The above-mentioned results reveal that two secreted proangiogenic proteins are mediated by ETV5, and accelerated angiogenesis occurs in CRCs with high expression of ETV5 (ETV5^+^ CRCs). ETV5-mediated secretion of CCL2 may be the critical mechanism underlying Bevacizumab resistance in CRC. According to endothelial tube formation analyses, the increased tubular formation of HUVECs with combined treatment of recombinant human CCL2 and VEGFA protein in the ETV5 knock-down group was more evident than that of the single treatment group in HT29 cells. Moreover, the combination of Bevacizumab and an antiCCL2 antibody more dramatically reversed the tubular formation of HUVECs induced by ETV5 upregulation than that observed with individual treatments in RKO cells (Fig. 6a). Meanwhile, following treatments using the supernatant of CRC cells and antibodies/recombinant proteins, the same trend was observed for pVEGFR2, pAKT, and pP38 in HUVECs (Fig. 6b). CAM assays were also performed and the results indicated that the HT29/shETV5 group treated with a combination of recombinant human CCL2, and VEGFA protein exhibited a higher level of angiogenesis than that exhibited by those groups exposed to individual treatments of recombinant human CCL2 or VEGFA protein. Similarly, the RKO/ETV5 group that was treated with a combination of Bevacizumab and antiCCL2 antibody exhibited a lower level of angiogenesis than that exhibited by the groups exposed to individual treatments with Bevacizumab or antiCCL2 antibody (Fig. 6c). In vivo, the tumors in the RKO/ETV5+Bevacizumab/antiCCL2 group grew slower than those in the RKO/ETV5+Bevacizumab or RKO/ETV5+antiCCL2 groups (Fig. 6d). Thirty days later, the subcutaneous tumors were removed for measurement. The sizes of the tumors in the RKO/ETV5+Bevacizumab/antiCCL2 group were also smaller than those of the tumors in the RKO/ETV5+Bevacizumab or RKO/ETV5+antiCCL2 groups (Fig. 6e). Additionally, the expression of CD31 and ki67 was lower in the groups treated with Bevacizumab and antiCCL2 antibody than that in the groups exposed to individual treatments with Bevacizumab or antiCCL2 antibody (Fig. 6f).

### ETV5, VEGFA, and CCL2 exhibit positive expression correlations with angiogenesis and are positively correlated with poor prognosis in CRC

**Fig. 7:**
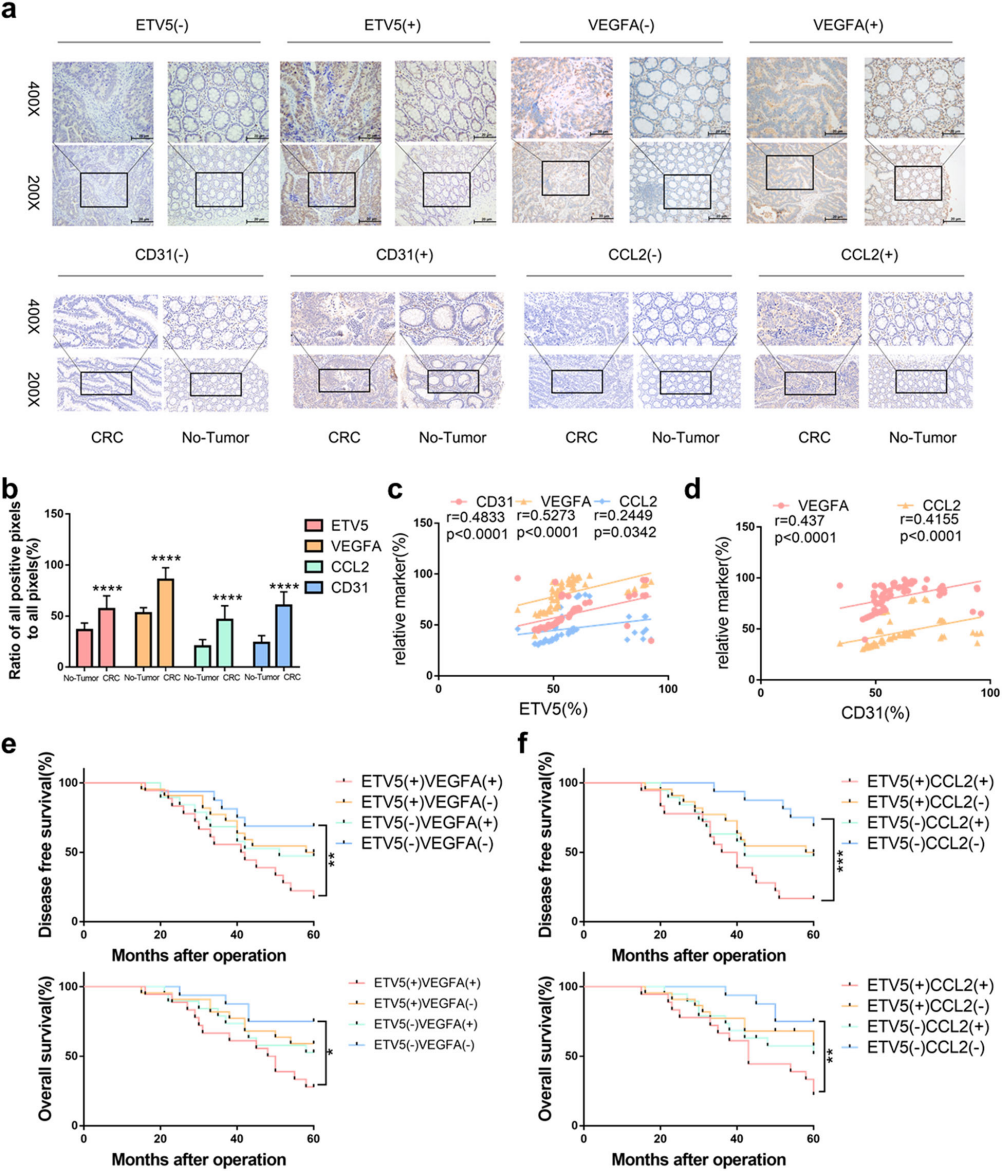
ETV5, VEGFA, and CCL2 show positive expression correlations with angiogenesis and are positively correlated with poor prognosis in CRC. **a** Representative images of IHC staining for ETV5, VEGFA, CCL2, and CD31 in the 75-patient cohort. **b** Expression of ETV5, VEGFA, CCL2, and CD31 was up-regulated in CRC tissues compared to levels in normal tissues (all *p* < 0.0001). **c** Expression of ETV5 was positively related to expression of VEGFA (*p* < 0.0001), CCL2 (*p* = 0.0342), and CD31 (*p* < 0.0001) in CRC tissues. **d** VEGFA (*p* < 0.0001) and CCL2 (*p* = 0.0002) were significantly associated with CD31 in CRC tissues. **e**, **f** Disease-free survival (DFS) and overall survival (OS) curves of the 75 patients in the cohort, as stratified by ETV5 and VEGFA expression patterns or by ETV5 and CCL2 expression patterns. *p < 0.05; **p < 0.01, ***p < 0.001, ****p < 0.0001.

**Fig. 8:**
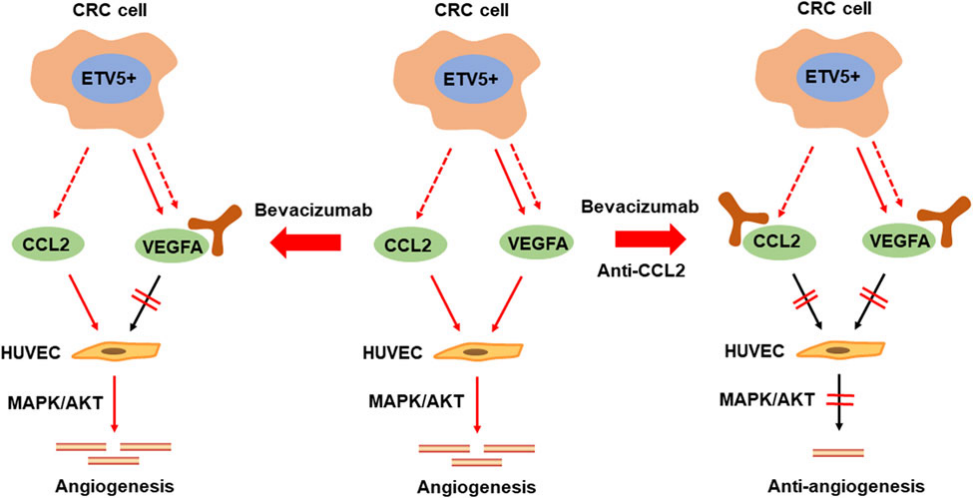
Schematic of ETV5-mediated anti-VEGF therapy resistance in ETV5^+^ CRC. In CRC, ETV5 could promote angiogenesis via the secretion of VEGFA and CCL2. When ETV5^+^ CRC was treated with Bevacizumab, paracrine CCL2 could induce angiogenesis by activating the MAPK and AKT pathways in human umbilical vein endothelial cells, ultimately resulting in Bevacizumab resistance. Therefore, a combination using Bevacizumab and antiCCL2 treatment could synergistically suppress angiogenesis by simultaneously neutralizing ETV5-induced VEGFA and CCL2 in CRC, and this combined therapy might provide promising anti-angiogenic strategy for ETV5^+^ CRCs.

Using IHC staining, we detected the protein levels of ETV5, VEGFA, CCL2, and CD31 in 75 paired CRC and normal tissues (Fig. 7a). Expression levels of all these proteins were upregulated in CRC tissues compared to levels in normal tissues (Fig. 7b). We observed a positive correlation between ETV5 expression and VEGFA (*r* = 0.6089, *p* < 0.0001, Fig. 7c), CCL2 (*r* = 0.2449, *p* = 0.0342, Fig. 7c), and CD31 (*r* = 0.4833, *p* < 0.0001, Fig. 7c) expression. Furthermore, VEGFA (*r* = 0.4370, *p* < 0.0001, Fig. 7d) and CCL2 (*r* = 0.4155 *p* = 0.0002, Fig. 7d) were also significantly related to CD31 expression in CRC tissues. Patients with tumors that were positive for both ETV5 and VEGFA (Fig. 6e) or both ETV5 and CCL2 exhibited the worst OS and DFS (Fig. 7f). Mechanistically, ETV5 promoted CRC angiogenesis through increased secretion of VEGFA and CCL2. Despite receiving Bevacizumab treatment, angiogenesis in ETV5^+^ CRCs was not effectively blocked due to the presence of another proangiogenic factor (CCL2 that was also induced by ETV5, and this factor could promote angiogenesis by activating the MAPK and AKT pathways in HUVECs. Therefore, additional antiCCL2 treatment may provide a promising method to overcome Bevacizumab resistance by strongly inhibiting angiogenesis in CRC (Fig. 8).

## Discussion

ETV5 leads to tumor initiation, progression, and metastasis by governing numerous biological processes^31,32^, including cell cycle control, differentiation, proliferation, apoptosis, tissue remodeling, and angiogenesis^10^. The molecular stabilization and upregulation of ETV5 function to maintain homeostasis and carcinogenesis in breast and prostate cancer^32,33^. Furthermore, ETV5 has been reported to directly influence the transcription of MMP2 and TIMP to modify tumor growth^19,34^. In CRC, ETV5 was demonstrated to promote angiogenesis through PDGF-BB induced VEGFA in CRC^19^. In the present study, we further revealed that ETV5 directly promoted transcription of the pro-angiogenic factor VEGFA in CRC. Studies have reported that cancers with high levels of VEGFA are particularly sensitive to VEGFA inhibition, and these include liver cancer, sarcoma, and breast cancer^26–28^. Based on the observation that ETV5 could strongly induce VEGFA expression in CRC, we speculated that the ETV5^+^ CRCs would be extremely sensitive to VEGFA. However, when we exogenously expressed ETV5 in CRC cells, the tumors were resistant to antiVEGF therapy (Bevacizumab). It is possible that a paracrine method of activation may occur during the course of medical treatment^22,35,36^. Bevacizumab is a molecular-targeted drug that specifically binds to and neutralizes human VEGFA to inhibit VEGF signaling pathway^37^. Although Bevacizumab can be used to treat metastatic colorectal cancer^38,39^, Bevacizumab resistance limits its therapeutic efficacy. Our results indicated that ETV5 might provide a useful biomarker to assess Bevacizumab resistance in CRC.

To explore the potential mechanisms involved in Bevacizumab resistance in CRC, we used TCGA database to perform ETV5-related GSEA analysis. We found that ETV5 expression was significantly associated with the activation of a chemokine signaling pathway, and this could also contribute to angiogenesis in a number of cancers^29,30^. Of the significantly enriched genes, four genes were proangiogenic factors, including CXCL11, CXCL5, CCL2, and CCL13^19^. Among these, only CCL2 was markedly attenuated by ETV5 knock-down in CRC cells. CCL2 was identified as another proangiogenic factor that was induced by ETV5 in CRC. CCL2/CCR2 chemokine signaling was demonstrated to promote breast cancer progression by inducing angiogenesis^20^. Although previous studies reported that CCL2 could be transactivated by several transcriptional factors such as NF-κB, STAT3, STAT1, Twist1, and ETS1^40^, we confirmed in the present study that ETV5-activated STAT3 could enhance CCL2 transcription in CRC. Using an ELISA assay, we found that attenuation of VEGFA did not affect ETV5-mediated CCL2 secretion, and antiCCL2 treatment also did not influence ETV5-mediated VEGFA secretion in CRC cells. These results suggested that VEGFA and CCL2 might act as two parallel signals to induce angiogenesis in ETV5^+^ CRCs. Further experiments confirmed that ETV5-mediated CCL2 secretion by CRC cells promoted Bevacizumab resistance in a manner that involved a paracrine activation effect in HUVECs. Therefore, when the ETV5^+^ CRCs received Bevacizumab treatment, the secretion of CCL2 induced by upregulation of ETV5 continued to activate angiogenesis-related pathways, such as the PI3K/AKT and p38/MAPK signaling pathways in HUVECs and this resulted in persistent angiogenesis^8^. Our results confirmed that ETV5-mediated secretion of CCL2 played a crucial role in Bevacizumab resistance.

Single drug treatment often results in drug resistance in cancers, and combined treatments have been demonstrated to elicit improved effects^41,42^. Our results indicated that both VEGFA and CCL2 participated in angiogenesis in ETV5^+^ CRCs. Additionally, our finding that CRCs with high expression of ETV5/VEGFA or ETV5/CCL2 showed inferior prognosis reinforced the critical roles of VEGFA and CCL2 in CRC progression. Treatments targeting CCL2 may provide a promising method to overcome antiVEGF therapy resistance of ETV5^+^ CRCs. In a phase 2 study, carlumab (CNTO 888), a human monoclonal antibody against CC-chemokine ligand 2 (CCL2), exhibited antitumor activity when used as a single agent in metastatic castration-resistant prostate cancer^43^. Based on this, we performed a combined treatment using Bevacizumab and antiCCL2 in nude mice. The combined treatment exhibited an improved efficiency in inhibiting tumor growth and angiogenesis than that exhibited by individual drug treatments. In HUVECs, phosphorylation of AKT and p38 could promote tubular formation in cancers^44,45^. Mechanistically, in the present study, the combined treatment exhibited an improved inhibitory efficacy in downregulating the angiogenesis-related PI3K/AKT and p38/MAPK pathways, than that exhibited by individual drug treatments in HUVECs. Owing to the complicated signaling pathway and interaction network involving multiple proteins, single drug treatment often achieve only limited effects in cancers, and more rational combined treatment is required^46,47^. Feig et al. reported that the compound AMD3100, which targets CXCL12/CXCR4 signaling, could overcome antiCTLA4 and antiPD-1 treatment resistance by depleting carcinoma-associated fibroblasts in pancreatic cancer^48^. Savino et al. found that combined targeting of CCR2 and the ERK pathway might provide a promising therapeutic strategy for the treatment of Kaposi sarcoma, where these treatments involve the inhibition of angiogenesis and subsequent tumor growth. Thus, we conclude that treatments targeting CCL2/CCR2 may provide an effective method to reverse Bevacizumab resistance in ETV5^+^ CRC.

In conclusion, our data revealed the role of ETV5 as a novel biomarker for Bevacizumab treatment in CRC. ETV5-mediated CCL2 promotes Bevacizumab resistance, and a combination of Bevacizumab and antiCCL2 treatment should be considered as a promising anti-angiogenic therapeutic strategy for ETV5^+^ CRCs.

## Supplementary information

Supplementary Figure 1

Supplementary Figure Legends

## Data Availability

All the data generated or analyzed during this study are included in this published article.
