# Correction: Targeting tumor cell-derived CCL2 as a strategy to overcome Bevacizumab resistance in ETV5^+^ colorectal cancer

**DOI:** 10.1038/s41419-020-03208-z

**Published:** 2020-11-23

**Authors:** Haoran Feng, Kun Liu, Xiaonan Shen, Juyong Liang, Changgang Wang, Weihua Qiu, Xi Cheng, Ren Zhao

**Affiliations:** 1 Department of General Surgery, Ruijin Hospital, School of Medicine, Shanghai Jiao Tong University, 200025 Shanghai, China; 2 Shanghai Institute of Digestive Surgery, Ruijin Hospital, School of Medicine, Shanghai Jiao Tong University, 200025 Shanghai, China; 3 Department of General Surgery, Ruijin Hospital North, School of Medicine, Shanghai Jiao Tong University, 201800 Shanghai, China; 4 Division of Gastroenterology and Hepatology, Renji Hospital, School of Medicine, Shanghai Jiao Tong University, 145 Middle Shandong Road, 200001 Shanghai, China

**Keywords:** Cancer therapeutic resistance, Chemotherapy

Correction to: *Cell Death and Disease*

10.1038/s41419-020-03111-7 published online 24 October 2020

The original version of this Article was updated shortly after publication following a mistake at proof stage, which resulted in the wrong version of Figure 1 being uploaded. The figure has now been replaced with the correct version in both the PDF and HTML versions of the Article.

**Fig. 1:**
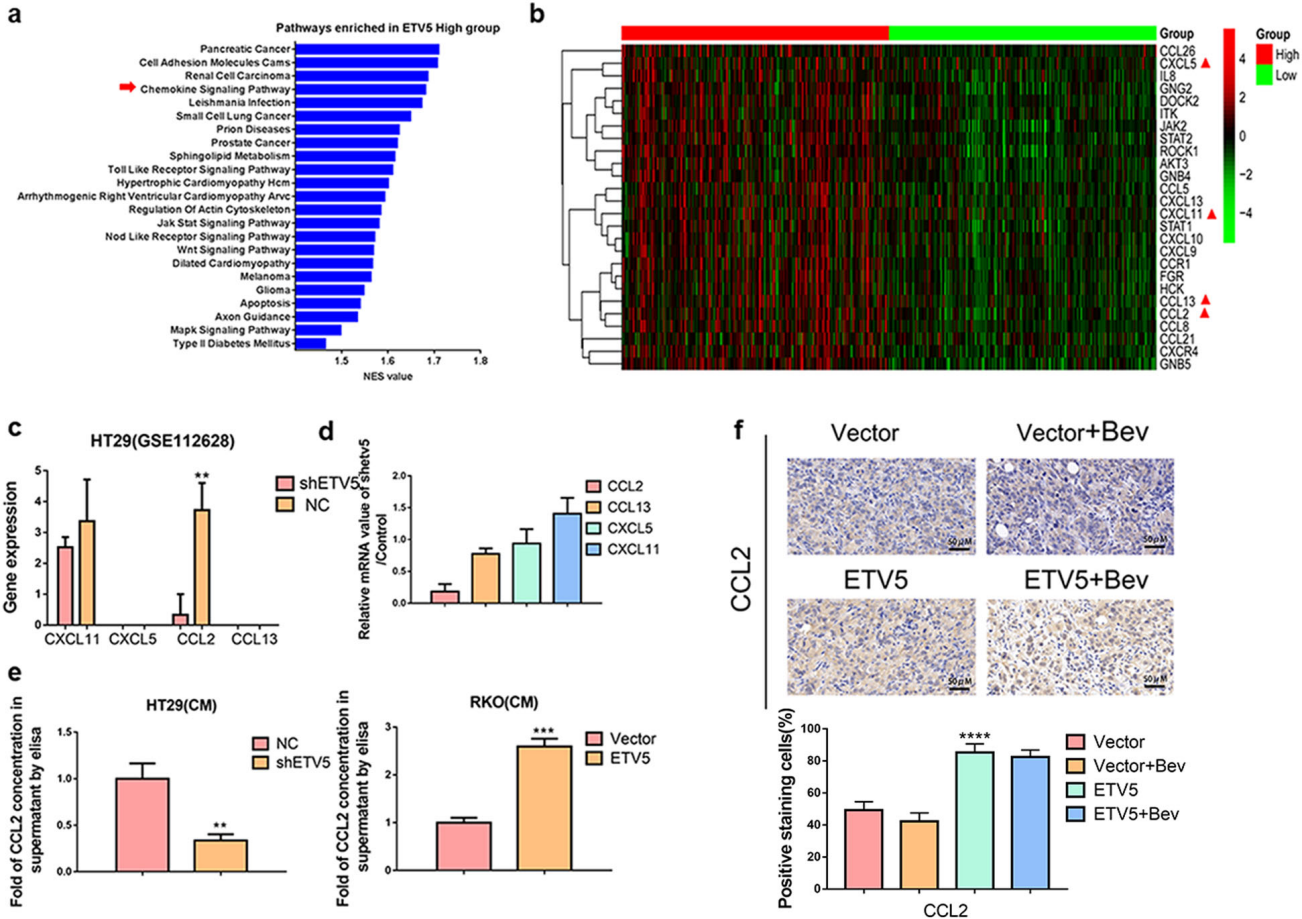
..

