# Supplementary figures and images for: Targeting tumor cell-derived CCL2 as a strategy to overcome Bevacizumab resistance in ETV5+ colorectal cancer
Source: Cell Death Dis. 2020 Oct 24;11(10):916. doi: 10.1038/s41419-020-03111-7 (PMC7585575; doi:10.1038/s41419-020-03111-7)

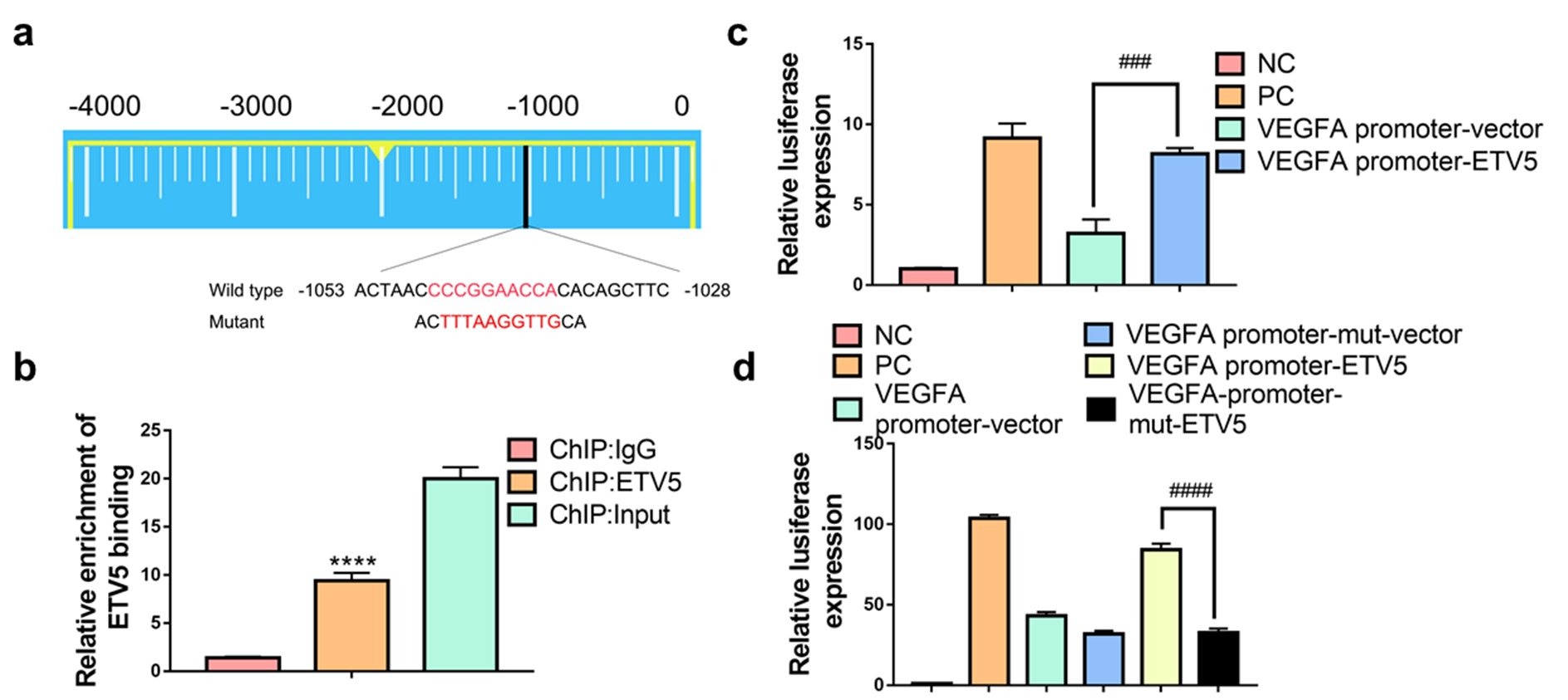

Supplement: Supplementary file 1 — Supplementary Figure 1 [file 41419_2020_3111_MOESM1_ESM.tif]
