# Supplementary material for: Targeting tumor cell-derived CCL2 as a strategy to overcome Bevacizumab resistance in ETV5+ colorectal cancer
Source: Cell Death Dis. 2020 Oct 24;11(10):916. doi: 10.1038/s41419-020-03111-7 (PMC7585575; doi:10.1038/s41419-020-03111-7)
Supplement: Supplementary file 2 — Supplementary Figure Legends [file 41419_2020_3111_MOESM2_ESM.docx]

**Supplementary Figure 1(Figure S1). ETV5 directly binds to the VEGFA promoter.** **a** Diagram of the VEGFA promoter, where the black marker indicates the ETV5-binding sites. **b** ChIP was performed using an anti-ETV5 antibody in RKO cells to analyze ETV5 binding to the VEGFA promoter. RT-PCR experiments were performed using primers against the indicated area in the VEGFA promoter, and the indicated region showed significant enrichment compared to that of the control. **c** and **d** Analysis of luciferase activity of the wild type (WT) VEGFA promoter-driven luciferase reporter in RKO cells or of the WT and mutant (MUT) VEGFA promoter-driven luciferase reporters in ETV5-overexpressing cells. NC: negative control. PC: positive control. Data are presented as mean ±SD of three independent experiments. “*” represents in comparison with the control. *****p*<0.0001, ^###^*p<*0.001, ^####^*p<*0.0001.
